# Variable versus conventional lung protective mechanical ventilation during open abdominal surgery: study protocol for a randomized controlled trial

**DOI:** 10.1186/1745-6215-15-155

**Published:** 2014-05-02

**Authors:** Peter M Spieth, Andreas Güldner, Christopher Uhlig, Thomas Bluth, Thomas Kiss, Marcus J Schultz, Paolo Pelosi, Thea Koch, Marcelo Gama de Abreu

**Affiliations:** 1 Pulmonary Engineering Group, Department of Anesthesiology and Intensive Care Medicine, University Hospital Dresden, Technische Universität Dresden, Fetscherstrasse 74, 01307 Dresden, Germany; 2 Department of Intensive Care Medicine, Academic Medical Center at the University of Amsterdam, Meidbergdreef 9, 1105 AZ Amsterdam, The Netherlands; 3 IRCCS AOU San Martino-IST, Department of Surgical Sciences and Integrated Diagnostics, University of Genoa, Largo Roseanna Benzi 8, 16132 Genoa, Italy

**Keywords:** Mechanical ventilation, Variable ventilation, General anesthesia, Abdominal surgery, Lung protective ventilation, Pulmonary complications

## Abstract

**Background:**

General anesthesia usually requires mechanical ventilation, which is traditionally accomplished with constant tidal volumes in volume- or pressure-controlled modes. Experimental studies suggest that the use of variable tidal volumes (variable ventilation) recruits lung tissue, improves pulmonary function and reduces systemic inflammatory response. However, it is currently not known whether patients undergoing open abdominal surgery might benefit from intraoperative variable ventilation.

**Methods/Design:**

The PROtective VARiable ventilation trial (‘PROVAR’) is a single center, randomized controlled trial enrolling 50 patients who are planning for open abdominal surgery expected to last longer than 3 hours. PROVAR compares conventional (non-variable) lung protective ventilation (CV) with variable lung protective ventilation (VV) regarding pulmonary function and inflammatory response. The primary endpoint of the study is the forced vital capacity on the first postoperative day. Secondary endpoints include further lung function tests, plasma cytokine levels, spatial distribution of ventilation assessed by means of electrical impedance tomography and postoperative pulmonary complications.

**Discussion:**

We hypothesize that VV improves lung function and reduces systemic inflammatory response compared to CV in patients receiving mechanical ventilation during general anesthesia for open abdominal surgery longer than 3 hours. PROVAR is the first randomized controlled trial aiming at intra- and postoperative effects of VV on lung function. This study may help to define the role of VV during general anesthesia requiring mechanical ventilation.

**Trial registration:**

Clinicaltrials.gov NCT01683578 (registered on September 3 3012).

## Background

General anesthesia promotes pulmonary atelectasis [1] and this effect is further enhanced during open abdominal surgery [2], mainly due to the use of muscle paralysis. Indeed, with relaxation of the diaphragm, the intra-abdominal pressure is transmitted to the intrathoracic cavity, promoting compression of the lung parenchyma and resulting in more atelectasis. Atelectasis and the decrease in pulmonary gas volume can persist during the postoperative period. It has been shown that forced vital capacity (FVC) and other variables of lung function are reduced for up to three hours postoperatively compared to pre-operative values [3]. It has been shown that noninfectious pulmonary complications like atelectasis or pleural effusions may favor the development of pneumonia [4], and increased bacterial translocation has been found from atelectatic lung regions [5]. The presence of atelectasis may promote the development of ventilator-associated lung injury due to cyclic collapse and re-opening of atelectatic lung regions [6]. Consequently, atelectasis formation is associated with increased length of stay in the recovery room and also with increased admission to the intensive care unit [7,8].

The application of positive end-expiratory airway pressure (PEEP) combined with lung recruitment maneuvers has been shown to reduce atelectasis and improve lung function [9,10]. However, recruitment maneuvers and high levels of PEEP carry the risk of barotrauma and deterioration of hemodynamics, and do not represent standard of practice in general anesthesia.

Recent data suggest that variable ventilation may improve lung function without causing structural damage to the lungs or increasing pulmonary inflammatory response in the experimental [11] and clinical setting [12]. One important mode of action of variable ventilation is the recruitment of previously collapsed lung areas. Variable ventilation is not only able to induce lung recruitment [13], but also to keep alveoli open once they are recruited [14]. This mechanism is of special importance in the presence of low or moderate settings of positive end-expiratory pressure, as planned in the presented trial. Variable ventilation mimics some aspects of the physiological ventilation pattern of healthy, spontaneously breathing individuals, but so far it is not known if variable ventilation is able to improve postoperative pulmonary function and reduce systemic pro-inflammatory response after surgery.

Recently, it has been demonstrated that a lung protective ventilation approach for general anesthesia can improve lung function and decrease postoperative pulmonary complications after open abdominal surgery [9]. The PROtective VARiable ventilation trial (‘PROVAR’) compares conventional (non-variable) lung protective ventilation (CV) and variable lung protective ventilation (VV) regarding pulmonary function and systemic inflammatory response. We hypothesized that VV improves lung function, redistributes ventilation toward gravity-dependent lung areas and reduces systemic inflammatory response compared to CV during controlled mechanical ventilation without spontaneous breathing activity in patients planned for open abdominal surgery expected to be longer than 3 hours.

## Methods/Design

### Objectives and design

PROVAR is an investigator-initiated, single center, randomized controlled, two-arm trial comparing CV and VV in patients receiving mechanical ventilation during general anesthesia for open abdominal surgery.

The study protocol is approved by the institutional review board of the Medical Faculty and the University Hospital Dresden, Germany (EK 174052011) and has been registered at clinicaltrials.gov (NCT01683578). PROVAR is conducted in accordance with the Declaration of Helsinki and the principles of good clinical practice.

**Figure 1:**
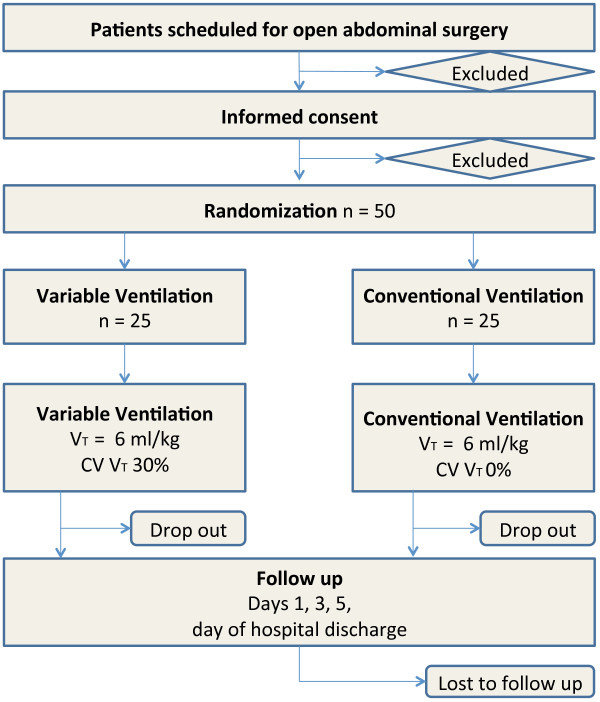
**CONSORT diagram for the PROVAR trial.** V_T_, tidal volume; CV V_T,_ coefficient of variation of tidal volume.

PROVAR tests the hypothesis that intraoperative VV is associated with improved pulmonary function and reduced systemic inflammatory response compared to CV in patients planned for open abdominal surgery. Primary endpoint is the FVC on the first postoperative day. Secondary endpoints are plasma concentrations of interleukin (IL)-6, IL-8 and tumor necrosis factor alpha (TNF-α); partial pressure of oxygen in capillary blood (PcO_2_) on the first postoperative day; peak expiratory flow (PEF) on the first postoperative day; forced expiratory volume in the first second (FEV_1_) on the first postoperative day; oxygenation (PaO_2_/F_I_O_2_) during surgery; changes of spatial distribution of ventilation measured by electrical impedance tomography (EIT); amount of atelectasis measured with magnetic resonance imaging (MRI) on the first postoperative day; and occurrence of postoperative pulmonary complications defined by the modified clinical pulmonary infection score (mCPIS) [9]. Figure 1 shows the CONSORT diagram of PROVAR.

### Screening, enrollment, randomization and blinding

Investigators screen consecutive patients planned for open abdominal surgery with an expected duration >3 h at the University Hospital Dresden, Germany during regular anesthesiological preoperative evaluations. The screening started in September 2012, and a recruitment time of 24 months has been defined based on the incidence of major abdominal surgery and patient characteristics at our hospital. Demographic data of screened patients are recorded regardless of whether they meet the enrollment criteria. Informed consent is obtained from all patients prior to surgery. All screened patients are reviewed with respect to inclusion and exclusion criteria as summarized in Table 1.

**Table 1:** Inclusion and exclusion criteria for the PROVAR trial

**Inclusion**	**Exclusion**
Patients scheduled for elective open abdominal surgery with expected duration >3 h	Chronic lung disease, except COPD GOLD stage I and II and untreated bronchial asthma
ASA class 2 and 3	Body mass index >40
Expected extubation in the OR	Hypersensitivity or allergy against one of the drugs administered during the study or against drugs with similar chemical structure
Informed consent to participate in the study signed by the patient	Participation of the patient in another clinical trial within the last 4 weeks
	History of substance abuse or any other mental status possibly affecting informed consent
	Pregnancy or breastfeeding
	Women in an age range of possible pregnancy if not:
	• Postmenopausal (12 months of amenorrhea or 6 months of amenorrheawith serum FSH >40 lU/ml)
	• Postoperative (6 weeks after bilateral ovariectomy with or without hysterectomy)
	• Regular and correct use of contraceptives with failure rate of <1% per year
	• Sexual inactivity
	• Vasectomy of sexual partner
	Suspected low patient compliance
	Contraindication for MRI exams
	Mechanical ventilation within the last 30 days

ASA, American Society of Anesthesiologists; OR, operating room; COPD, chronic obstructive pulmonary disease; GOLD, global initiative for chronic obstructive lung disease; FSH, follicle-stimulating hormone; MRI, magnetic resonance imaging.

Randomization is performed by random block randomization with fixed 1:1 randomization ratios within blocks of 2 × 10, 3 × 6 and 3 × 4 patients using closed envelopes.

Blinding is achieved by means of a modified triple blind design with the patients and postoperative investigators blinded to the treatment and the intraoperative investigator blinded to the pre- and postoperative measurements.

### Intervention

After randomization, patients will be ventilated either with CV or VV, respectively. Both modes are performed with the same ventilator (EVITA XL4Lab, Dräger Medical, Lübeck, Germany) in a volume-controlled mode with a fraction of inspired oxygen (F_I_O_2_) of 0.35, PEEP of 5 cmH_2_O, tidal volumes (V_T_) of 8 ml/kg and an I:E ratio of 1:1. Respiratory rate is adjusted to achieve normocapnia (4.6 to 6.0 kPa). While V_T_ is kept constant during CV, it varies randomly on a breath-by-breath basis during VV. In VV, the mean V_T_ is kept at 8 ml/kg, and the distribution of values follows a Gaussian distribution with coefficient of variation at 30%, as described by our group in detail elsewhere [11]. Such mechanical ventilation mode is accomplished by computer remote control of the ventilator, which is allowed by the manufacturer for clinical use. Table 2 shows the ventilator settings. In case of desaturation (SpO_2_ < 94% for more than one minute) a standardized rescue maneuver is implemented in the protocol. After exclusion of nonventilatory causes, the following interventions will be consecutively performed: 1) a stepwise increase of F_I_O_2_ in steps of 10% up to 100%; 2) a stepwise increase of PEEP in steps of 1 cmH_2_O up to 12 cmH_2_O; and 3) a recruitment maneuver with P_aw_ 40 cmH_2_O for 20s. In case of malfunction or technical problems with the remote computer control during VV, the computer will be disconnected and the ventilator will be switched back to CV. In case of any ventilatory or other medical problems, interventions will be performed based on the discretion of the anesthesiologist in charge of the patient according to clinical standards and will be documented in the case report form accordingly.

**Table 2:** Intraoperative ventilator settings for the PROVAR trial

	**Conventional ventilation**	**Variable ventilation**
**F**_**I**_**O**_**2**_	0.35	0.35
**PEEP**	5 cmH_2_O	5 cmH_2_O
**V**_**T**_	8 ml/kg	Mean value of 8 ml/kg, breath by breath variability with a CV V_T_ of 30%
**RR**	Adjusted according normocapnia (4.6-6.0 kPa)	Adjusted according normocapnia (4.6 to 6.0 kPa)
**I:E**	1:1	1:1
**Flow**	30 L/min, adjusted in case of flow limitation	30 L/min, adjusted in case of flow limitation
**P**_**max**_	40 cmH_2_O	40 cmH_2_O

F_I_O_2,_ fraction of inspired oxygen; PEEP, positive end-expiratory pressure; V_T_, tidal volume; RR, respiratory rate; I:E, ratio of inspiratory to expiratory time; Flow, air flow; P_max_, maximum of inspiratory pressure; CV V_T_, coefficient of variation of tidal volume.

### Time course of interventions/study protocol

At day 0 (visit 1), informed consent is obtained, demographic data are recorded and spirometry, EIT measurements and capillary blood gas analysis performed to retrieve baseline data. Additionally, potentially fertile female patients are submitted to a pregnancy test.

Visit 2 is performed on the day of surgery. A preoperative MRI of the lungs is performed prior to premedication. Preparation for anesthesia and surgery is done according to standard of care. Anesthesia induction and maintenance is standardized according to the following protocol: all weight-derived calculations are based on ideal body weight defined as 50 or 45.5 (male or female, respectively) + 0.91*(height - 152.4): Anesthesia induction is performed with continuous infusion of 0.5 μg/kg/h remifentanil and a bolus of 1 to 2 mg/kg propofol. Neuromuscular blockade to facilitate endotracheal intubation is achieved by bolus administration of 0.2 mg/kg cisatracurium. Maintenance of anesthesia is monitored by bispectral index (BIS level 40 to 60) and is achieved by continuous infusion of 5 mg/kg/h propofol, 0.2 to 0.3 μg/kg/h remifentanil and 0.09 mg/kg/h cisatracurium. Neuromuscular blockade is checked hourly by muscle relaxometry using the train of four ratio aiming at values of 0 to 1. During induction of anesthesia, an electrolyte solution is administered at a rate of 500 ml/h and reduced thereafter to a maintenance rate of 300 ml/h. Noradrenaline can be infused if necessary to ensure mean arterial blood pressure levels >60 mmHg. After induction and prior to the start of surgery, postinduction measurements are performed and patients are then randomly assigned to mechanical ventilation with CV or VV. EIT measurements and blood plasma samples are obtained postinduction, as well as after surgical wound closure (postclosure) before the end of anesthesia. Arterial blood gases are measured hourly throughout surgery and hemodynamics, as well as spirometric data, are recorded from the monitoring system and the ventilator, respectively. Amounts of infused drugs and fluids, as well as blood loss and urine output, are recorded. Visit 3 takes place on the first postoperative day. Spirometry, lung MRI, EIT, plasma blood sampling and capillary gasometry are performed, and adverse, as well as severe adverse, events (AEs, SAEs) are recorded.

Visit 4 is performed on the third postoperative day. Spirometry, EIT, plasma blood sampling and capillary gasometry are obtained, and AEs and SAEs recorded.

Visit 5 takes place on the fifth postoperative day and includes spirometry, EIT, plasma blood sampling and capillary gasometry. AEs and SAEs will be recorded.

Visit 6 represents the final examination before hospital discharge. Spirometry, EIT, plasma blood sampling and capillary gasometry will be measured, and AEs and SAEs will be recorded.

### Technical aspects

For electrical impedance tomography (EIT), a PulmoVista® 500 (Dräger Medical, Lübeck, Germany) will be used. Files will be acquired over 2 min, with a frame rate of 20 Frames/s. Distribution of ventilation across the ventral-dorsal gradient will be determined from relative changes in electrical impedance as described elsewhere [15]. Briefly, images of the EIT device containing 32 × 32 pixels will be recorded at a rate of 20 frames/s during 2 min for offline analysis. Using a dedicated routine (EITa!), the highest and lowest limits of the area containing changes in the impedance (region of interest (ROI)) will be determined. The ROI will be divided into three zones with equal heights (ventral, central and dorsal) and the relative changes in impedance will be computed.

Magnetic resonance imaging (MRI) will be performed preoperatively and at the first postoperative day. Native MRI sequences using T2 (HASTE: TR/TE = 1000 ms/98 ms; 360 ms per slice) and T1 (VIBE: TR/TE = 5 ms/2 ms; 12 s per volume × 2) will be obtained during inspiratory breath hold. A radiologist blinded to the study groups will manually measure thickness of atelectasis and pleural effusions. Volumetric measurements of aerated and nonaerated lung tissue will be performed using manual segmentation and volumetric measurements using the OsiriX software package.

### Study dropouts

Participation in the trial is voluntary. A subject has the right to withdraw from the study at any time for any reason without any consequences for further medical treatment. Furthermore, both investigators, intraoperative as well as postoperative, have the right to terminate the participation of any subject at any time during the part of the study they are responsible for if the investigator deems it in the participant’s best interest. The reasons and circumstances for study discontinuation will be documented in the Case Report Form (CRF). All data are analyzed based on the intention-to-treat (ITT) principle.

### Statistics

Sample size calculation was based on a previous study using FVC as a measure of postoperative pulmonary function following general anesthesia [3]. In the present study, effect size was estimated as 1.086, alpha was defined as 0.05 and power as 0.95. Using a two-tailed Mann–Whitney-*U*-test, sample size calculation yielded 25 patients per group. Analysis was performed using GPower (Software Version 3.1.3, University of Düsseldorf, Germany).

Exploratory analysis will include mean and standard deviation for normally distributed variables. Non-normally distributed variables will be expressed by their medians and interquartile ranges. Categorical variables will be expressed as n (%). Parametrical or non-parametrical tests will be used as appropriate to analyze the data. *P* values for multiple comparisons will be adjusted according to the Bonferroni procedure.

In case of loss to follow-up or consent withdrawal from the trial, the causes will be reported. ITT and per protocol (PP) analysis will be conducted. For the intention-to-treat analysis, data will be processed for all trial patients in the groups in which they were randomized. The PP analysis will be performed as a secondary analysis if there are a considerable number of patients who do not receive study therapy or are lost to outcome assessment. Missing data will be handled by means of the last observation carried forward method.

### Study organization

All serious adverse events and all unexpected and related or possibly related adverse events will be reported to the internal review board of the Medical Faculty and the University Hospital Dresden. Regular checks for plausibility and protocol adherence will be done according to good clinical practice (GCP) guidelines. Two independent experts in the field that are not part of the study group serve on the study’s data safety and monitoring board (DSMB) to ensure adherence to the study protocol, quality of data collection and processing as well as safety issues related to the study.

## Discussion

The concept of VV has been extensively studied in recent years. A growing body of experimental evidence suggests the beneficial effects of variable ventilatory modes mimicking physiological variability of the respiratory system [11,13,16,17]. After the introduction of variable pressure-support ventilation (noisy PSV) by our group in 2008 [18], we could demonstrate that the beneficial effects of VV are not only limited to controlled mechanical ventilation but can also be applied during assisted spontaneous breathing [15,19]. Comparing biological variable ventilation and random (noisy) variability using equal amounts of variability, it has been demonstrated that both respiratory patterns yielded comparable effects on lung function. The authors conclude that the amount, but not the pattern, of respiratory variability is crucial for the success of VV [20]. It is worth noting that the variability administered during VV in this study is closely related to the physiological variability of the respiratory system [11,21,22] and has been found to be superior to higher or lower levels of variability in previous experimental studies [23].

Several mechanisms have been proposed to explain the beneficial effects of VV: 1) the *de novo* synthesis of surfactant proteins seems to be enhanced during VV [24,25]; 2) VV seems to recruit previously closed alveolar units [14,26]; 3) the concept of stochastic resonance may explain improvements in respiratory mechanics [17]; and 4) the beneficial effects on gas exchange may be explained by improved ventilation/perfusion matching [18,27].

In the only clinical trial performed with VV in patients undergoing general surgery so far, Boker and colleagues could demonstrate that biological variable ventilation improves gas exchange and respiratory mechanics compared to CV in patients undergoing abdominal aortic aneurysm repair [12]. Their results were confirmed by a small pilot study in eight critically ill patients [28]. The PROVAR study aims at closing the gap between preclinical data and initial clinical application by providing new insights into the perioperative physiological changes due to different intraoperative mechanical ventilation modalities.

This study protocol has several limitations that need to be addressed:

1. The duration of mechanical ventilation may be different across patients due to different lengths of surgery.

2. As part of the clinical routine some patients will receive thoracic epidural anesthesia, whereas others who have contraindications will not; therefore, thoracic epidural anesthesia will be implemented as a covariate in the statistical analysis.

3. Generalizability of the study results is critical since until today there is no commercially available ventilator or anesthesia machine able to perform VV.

4. The anesthesiologist in charge of the intraoperative part of the study cannot be blinded to the ventilator mode; we therefore decided to split personnel for intra- and perioperative analysis in order to have at least the perioperative staff blinded to the ventilator mode.

In conclusion, PROVAR is the first randomized controlled trial aiming at intra- and postoperative effects of VV on lung function. This study may help to define the role of VV during general anesthesia requiring mechanical ventilation.

## Trial status

Enrollment has begun.

## Abbreviations

AE: adverse event; CRF: case report form; CV: conventional lung protective ventilation; DMSB: Data Safety and Monitoring Board; EIT: electrical impedance tomography; FEV1: forced expiratory volume in the first second; FVC: forced vital capacity; GCP: good clinical practice; IL: interleukin; ITT: intention to treat; mCPIS: modified clinical pulmonary infection score; MRI: magnetic resonance imaging; PaO2/FIO2: ratio of arterial partial pressure of oxygen and inspired oxygen fraction; PcO2: partial pressure of oxygen in capillary blood; PEEP: positive end-expiratory airway pressure; PEF: peak expiratory flow; PP: per protocol; PROVAR: The PROtective VARiable ventilation trial; SAE: severe adverse event; TNF-α: tumor necrosis factor alpha; VV: variable lung protective ventilation.

## Competing interests

Drs. Spieth, Koch and Gama de Abreu were granted three patents on variable ventilation.

## Authors’ contributions

PMS participated in the design of the study, drafted the manuscript and serves as the study coordinator. AG participated in the design of the study and drafted the manuscript. CU drafted the manuscript. TB drafted the manuscript. TK drafted the manuscript. MJS participated in the design of the study and drafted the manuscript. PP participated in the design of the study and drafted the manuscript. TK participated in the design of the study and drafted the manuscript. MGA participated in the design of the study, drafted the manuscript and serves as the principal investigator. All authors read and approved the final manuscript.
